# Dripping, Jetting and Regime Transition of Droplet Formation in a Buoyancy-Assisted Microfluidic Device

**DOI:** 10.3390/mi11110962

**Published:** 2020-10-27

**Authors:** Chaoqun Shen, Feifan Liu, Liangyu Wu, Cheng Yu, Wei Yu

**Affiliations:** College of Electrical, Energy and Power Engineering, Yangzhou University, Yangzhou 225127, China; cqshen@yzu.edu.cn (C.S.); ffliu@microflows.net (F.L.); lywu@yzu.edu.cn (L.W.)

**Keywords:** microfluidic, droplet formation, buoyancy, interfacial tension

## Abstract

Buoyancy-assisted droplet formation in a quiescent continuous phase is an effective technique to produce highly monodispersed droplets, especially millimetric droplets. A comprehensive study combining visualization experiment and numerical simulation was carried out to explore the underlying physics of single droplet generation in a buoyancy-assisted microfluidic device. Typical regimes, including dripping and jetting, were examined to gain a deep insight into the hydrodynamic difference between the regimes. Particularly, the transition from dripping regime to jetting regime was investigated to give an in-depth understanding of the transitional behaviors. The effects of interfacial tension coefficient on the droplet size and formation regimes are discussed, and a regime diagram is summarized. The results show that oscillation of the interface in dripping regimes after detachment is caused by the locally accelerated fluid during the neck pinching process. Droplet formation patterns with the characteristics of both dripping regime and jetting regime are observed and recognized as the transitional regime, and the interface oscillation lasts longer than that in dripping regime, implying intensive competition between interfacial tension and inertial force. Reducing interfacial tension coefficient results in the dripping-to-jetting transition occurring at a lower flow rate of the dispersed phase. The regime diagram indicates that only the inertial force is the indispensable condition of triggering the transition from dripping to jetting.

## 1. Introduction

Monodispersed emulsions are favorable in various applications, including inertial confinement fusion (ICF) target fabrication [1,2], cosmetics [3], drug delivery [4,5,6] and biological assays [7,8,9]. During the past decades, producing uniform droplets with polydispersity lower than 10% [10,11,12] through microfluidics [13] has been proved a robust and efficient approach [14,15,16]. Unlike the “top-down” emulsification methods such as stirring [17] and ultrasonic emulsification [18], strong impact and agitation are avoided in the “bottom-up” fashioned microfluidic methods. Hence, precise control over the formation process of each individual droplet [19] can be achieved by microfluidics. Usually, in microfluidics [16,20], droplets are produced in a continuous fluid flow during which the development and break-up of the interface is controlled or dominated by the viscous force from the flowing continuous phase [3,21,22]. Based on different configurations of continuous fluid flow field, the typical microfluidic devices for producing droplets can be classified into four categories [16]: co-flowing [23], flow-focusing [24], T-junctions [25] and pipetting [26,27] and step emulsification [28].

In general, interfacial tension, viscous force from the continuous phase and inertial force from the dispersed phase dominate the formation of droplets in the microfluidic devices [29]. Meanwhile, buoyancy is often neglected. On the contrary, inertial force and buoyancy become the two major forces that drive the dispersed phase away from its inlet in the buoyancy-assisted microfluidic devices [30]. No active flow is required in the continuous phase, which makes the buoyancy-assisted system less complicated. For example, two syringe pumps are required to inject the continuous phase and dispersed phase separately to produce single droplets in most devices. Only one syringe pump is needed for the buoyancy-assisted device, and hence the control strategy of the flow rate is simplified as well. Buoyancy-assisted devices were widely studied before the 1990s [31,32,33,34]. Due to the upsurging of soft lithography and other advanced fabrication techniques, devices with more complicated configurations have been developed. Of special note are the flow-focusing devices, which became the most popular droplet, microbubble and particle makers in the past two decades [35,36,37,38].

Due to the capability of producing monodispersed millimetric droplets, which is essential in applications that require big droplets, such as fabricating ICF targets [39], buoyancy-assisted droplet formation has once again attracted intensive attention. Experimental studies show that both single [40] and double emulsions [41,42] can be produced by the buoyancy-assisted devices. By placing the tip of a microchannel vertically underneath water, Chaurasia et al. [40] examined the formation of hydrocarbon oil droplets in both DI water and surfactant-loaded water. A modified model considering the dynamic interfacial tension was proposed that improves the droplet size prediction compared to Tate’s law [43], and the size distribution of droplets produced in the jetting regime is dependent on break-up lengths. Recently, to further explore the capability of producing double emulsions in the buoyancy-assisted microfluidics, Che et al. [42] developed a simple method for double emulsion production. In this method, the inner droplets are produced in the first co-flowing droplet maker and then encapsulated into double emulsions by the middle fluid in the second droplet maker assisted by buoyancy. A high degree of control over the droplet sizes and the number of the inner droplets is achieved, showing great potential in chemical engineering at the millimeter scale.

Though numerous studies have been conducted exploring the performance of buoyancy-assisted microfluidic devices, most have been focused on experimental measurement and theoretical prediction based on force balancing. Particularly, the underlying mechanism of the transition behavior still lacks in-depth investigation, which is essential in formulating the control strategies during droplet formation. In this context, combined efforts of visualization experiment and numerical simulation were conducted to clarify the transition from dripping regime to jetting regime in the buoyancy-assisted microfluidics. Dripping and jetting regimes are examined thoroughly in this work. The regime showing characteristics of both dripping and jetting is recognized and named transitional regime. The influence of the interfacial tension coefficient in the regime transition is examined. Finally, the effect of both Weber number and Bond number are discussed and summarized in a regime diagram.

## 2. Experimental Setup

**Figure 1:**
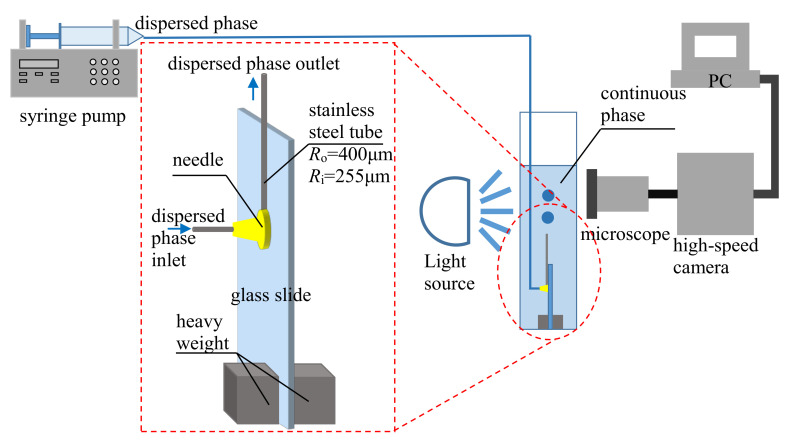
Experimental setup for droplet formation in buoyancy-assisted microfluidics.

In the buoyancy-assisted droplet formation system illustrated in Figure 1, the dispersed phase (n-octane, Sinopharm Chemical Reagent Co., Ltd., Shanghai, China) was injected through a stainless steel tube (outer radius *R*_o_ = 400 μm, inner radius *R*_i_ = 255 μm, Suzhou Lanbo Needle Co., Ltd., Suzhou, China) with the inlet of the tube placed beneath the surface of the continuous phase. The stainless steel tube was fixed on a piece of glass slide parallel to the long edge of the glass slide using epoxy glue. A needle was placed at the bottom end of the stainless steel tube and glued on the glass slide to inject the dispersed phase. Two pieces of heavy weight were glued at the bottom of the glass slide to prevent the device from floating. The whole device was placed at the bottom of a container filled with a stationary continuous phase. The dispersed phase was injected by a syringe pump (LSP02-1B, Baoding Longer Precision Pump), and the formation process of the droplet was recorded by a combined system of microscope (SZX7, Olympus, Tokyo, Japan) and high-speed camera (500K-M2, Photron, Tokyo, Japan). SDS (sodium dodecyl sulfate, Sinopharm Chemical Reagent Co., Ltd., Shanghai, China) was added to DI water to adjust the interfacial tension coefficient. The physical properties of all the fluids are listed in Table 1, where the column *σ* is the interfacial tension coefficient between SDS solution with different concentrations and n-octane measured using a ring method [44]. All the experiments were carried out in a lab with room temperature set at 20 ± 2 °C

## 3. Mathematical Model

**Figure 2:**
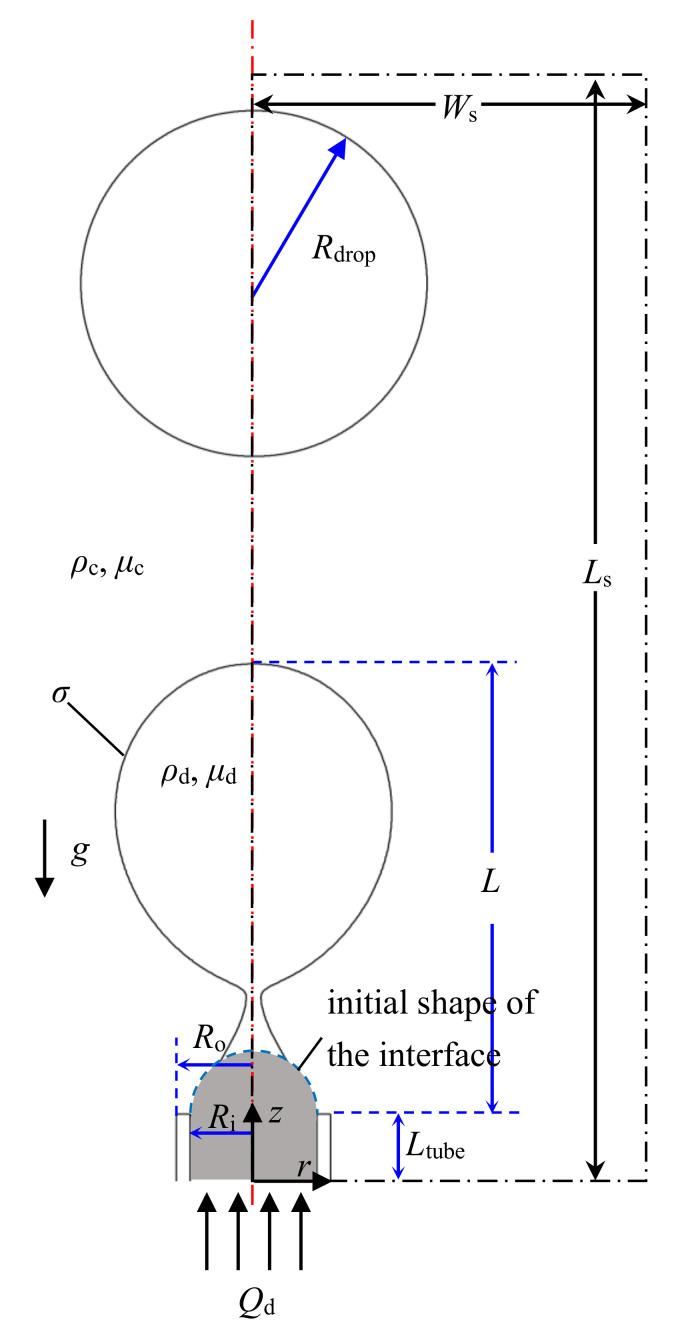
Schematic of the numerical simulation domain.

The numerical simulation was conducted in a computational domain with width of *W*_s_ = 5 mm and length of *L*_s_ = 20 mm as shown inside the dash dot lines in Figure 2. Since the droplet is formed in a circular tube, an axisymmetric configuration is utilized. Injection of the dispersed phase (density *ρ*_d_, viscosity *μ*_d_) into the stationary continuous phase (density *ρ*_c_, viscosity *μ*_c_) is through the inlet at the bottom with an inner diameter of *R*_i_ = 0.255 mm and an outer diameter of *R*_o_ = 0.4 mm, which is the same as in our experiment. The length of the tube is *L*_tube_ = 3.8 mm. The distance between the front of the interface to the tip of the tube is denoted as droplet length *L*. The droplets with diameter denoted as *R*_drop_ float away from the tip after detaching due to the buoyancy.

### 3.1. Governing Equations

The volume of fluid (VOF) method is used to capture the evolving interface in which different phases are distinguished according to volume fraction *a* as

(1) $$\left\{\begin{array}{c} a=0,\;\mathrm{the}\;\mathrm{computational}\;\mathrm{cell}\;\mathrm{does}\;\mathrm{not}\;\mathrm{contain}\;\mathrm{this}\;\mathrm{phase}\\ 0<a<1,\;\mathrm{the}\;\mathrm{computational}\;\mathrm{cell}\;\mathrm{contains}\;\mathrm{the}\;\mathrm{interface}\;\\ a=1,\;\mathrm{the}\;\mathrm{computational}\;\mathrm{cell}\;\mathrm{is}\;\mathrm{filled}\;\mathrm{with}\;\mathrm{this}\;\mathrm{phase}\; \end{array}\right.$$

Since there only one dispersed phase and one continuous phase are under consideration, the volume fractions of these two phases sum up to 1 in one computational cell:

(2) $$a_{c}+a_{d}=1$$

The transportation equations of the volume fractions are

(3) $$\frac{\partial a_{c}}{\partial t}+\nabla \cdot \left(U\cdot a_{c}\right)=0$$

(4) $$\frac{\partial a_{d}}{\partial t}+\nabla \cdot \left(U\cdot a_{d}\right)=0$$

where *t* is the flow time and the velocity *U* is solved from the continuum equation and momentum equation given as follows:

(5) ∇⋅U=0

(6) $$\frac{\partial U}{\partial t}+\nabla \cdot \left(UU\right)=-\frac{\nabla p}{\rho }+\nabla \cdot \frac{\mu }{\rho }\left[\nabla U+\nabla U^{T}\right]+f$$

in which *p* is pressure. The density *ρ* and viscosity *μ* are interpolated from the dispersed phase and continuous phase:

(7) $$\rho =a_{c}\rho_{c}+a_{d}\rho_{d}$$

(8) $$\mu =a_{c}\mu_{c}+a_{d}\mu_{d}$$

The source term *f* in Equation (6) comprises the buoyancy force and interfacial tension force:

(9) $$f=g+f_{sv}$$

where *g* is the gravitational acceleration. The interfacial tension *f*_sv_ is calculated by the continuum surface force model:

(10) $$f_{sv}=\sigma \kappa n\delta_{s}$$

where σ is the interfacial tension coefficient which is constant throughout the computational domain during each case simulated, *κ* is the curvature, *n* is the unit vector perpendicular to the interface and the Dirac equation *δ*_s_ is always 0 except in those computational cells that contain the interface.

The velocity inlet boundary condition is used for the inlet of the dispersed phase:

(11) $$U_{z}(0<r<R_{i})=U_{i}$$

Meanwhile, the pressure outlet boundary condition is applied at the boundary *z* = *L* as

(12) $${\left.p\right|}_{z=L}=0$$

Nonslip boundary conditions are applied at all wall boundaries. Initially, a semispherical dispersed phase droplet is attached at the tip of the tube as shown in Figure 2. The gray region inside the blue dashed line is filled with the dispersed phase.

Since no forced flow is implemented at the continuous phase, the forces that drive the droplet away from the outlet are buoyancy and inertial force, while the competing force that maintains the droplet’s attachment to the outlet is the interfacial tension. The relationship between the dominating forces can be expressed as dimensionless numbers Weber number *We* and Bond number *Bo* as [45]

(13) $$We=\frac{\rho_{d}Q_{d}u_{d}}{2\pi r_{w}\sigma }=\frac{inertial\;force}{interfacial\;tension}$$

(14) $$Bo=\frac{\Delta \rho gr_{w}^{2}}{\sigma }=\frac{buoyancy\;force}{interfacial\;tension}$$

where *ρ*_d_ is the density of the dispersed phase, *Q*_d_ is the flow rate the dispersed phase, *u*_d_ is the velocity of the dispersed phase, *r*_w_ is the wetting radius of the dispersed phase at the tip of the inlet tube and 𝛥*ρ* is the density difference between the dispersed phase and continuous phase.

### 3.2. Numerical Solution

The computational domain was discretized using quadrilateral cells with refinement along the axis. The commercial CFD software Fluent 6.3 was used as the solver. The Reynold number was less than 500 for all cases, and hence a laminar model was applied in our study. The second-order upwind scheme was used in discretizing the momentum equation, while the first-order upwind scheme was used in discretizing other equations. Coupling between the pressure and velocity was achieved through the semi-implicit method for pressure linked equations (SIMPLE) algorithm. A varying time step strategy based on global Courant number < 0.5 was adopted in order to reduce the time consumption of the computation. The convergence in one time step was achieved when the relative residuals were less than 1%.

**Figure 3:**
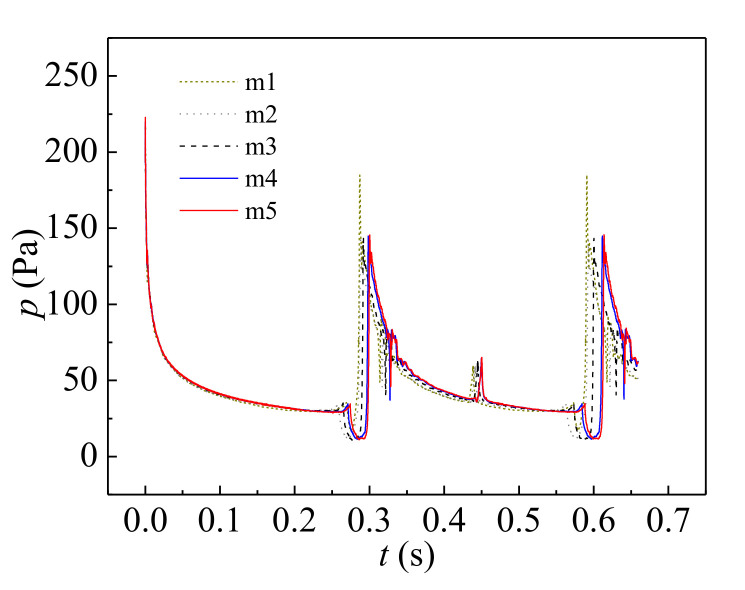
Pressure variation at location *z* = 5 mm, *r* = 0 mm calculated using different meshes.

A mesh independence test was carried out using five different meshes named Mesh1 (13,800 cells), Mesh2 (18,925 cells), Mesh3 (25,850 cells), Mesh4 (37,760 cells) and Mesh5 (50,548 cells), and the variation of the pressure at location *z* = 5 mm, *r* = 0 mm was considered (Figure 3). The simulation results using meshes M4 and M5 showed little difference. Hence, considering the balance between computational cost and accuracy, M4 was used in our study.

### 3.3. Case Validation

**Figure 4:**
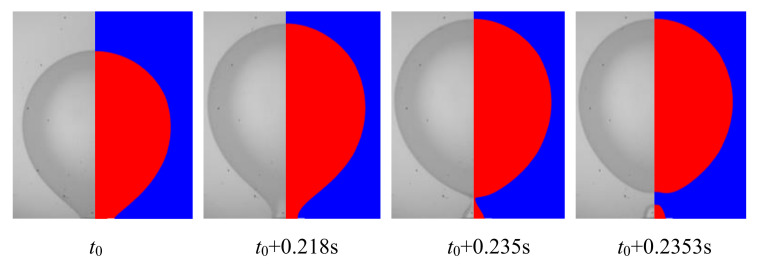
Case validation (*q*_d_ = 90 mL/s, *We* = 0.027, *Bo* = 0.0038).

The mathematical model was verified with our experimental results as shown in Figure 4. The interface reconstructed from the numerical simulation (left) was compared with the experiment snapshots (right) under the condition of n-octane droplets forming in DI water with *q*_d_ = 25 μL/s. The agreement between numerical and experimental results indicates that the model is accurate enough to predict the liquid–liquid multiphase flow in a buoyancy-assisted microfluidic device.

## 4. Results and Discussion

### 4.1. Dripping

**Figure 5:**
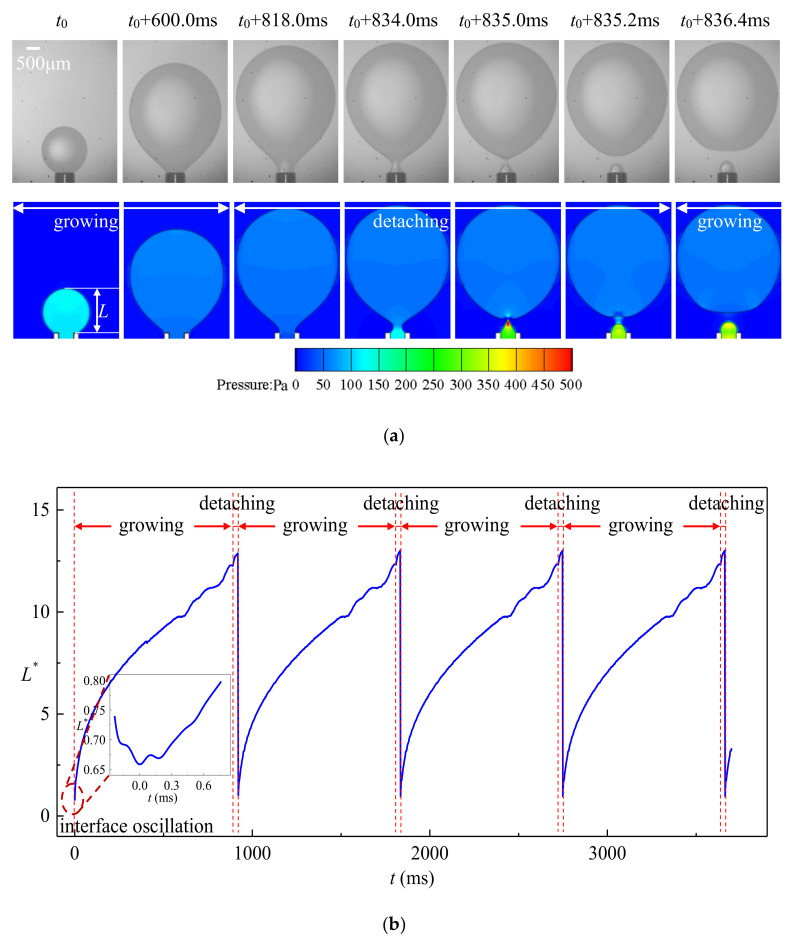
Typical dripping regime (*q*_d_ = 90 mL/h, *We* = 0.027, *Bo* = 0.0038): (**a**) experimental snapshots and numerical simulation; (**b**) variation of droplet length reconstructed from numerical simulation.

Dripping occurs when *Bo* + *We* < 1 and *We* < 0.5. The droplets are formed close to the tip of the tube with strict periodicity as shown in Figure 5 (*q*_d_ = 25 μL/s, *We* = 0.027, *Bo* = 0.0038). The dimensionless length of the droplet L^*^ is the ratio between the droplet length *L* and outer radius of the tube *R*_o_: L^*^ = *L*/*R*_o_. The formation process of the droplets can be divided into growing stage and detaching stage. Both experimental and numerical results indicate that more than 90% of the time during one droplet formation period is occupied by the growing stage. Since *We* << 1 and *Bo* << 1, the interfacial tension dominates the behavior of the fluids. The interface is approximately spherical during the early stage of growing since buoyancy is negligible. The volume of the droplet *V*_d_ ≈ π/6‧*L*^3^ and the variation of the droplet length d*L*/d*t* is proportional to *t*^−2/3^ as shown in Figure 5b. With the dispersed phase injected continuously, the volume of the droplets grows such that the buoyancy drives the dispersed phase towards the flow direction that the spherical shape of the droplets is derived from. The wetting ratio of the dispersed phase also decreases from *R*_o_ to *R*_i_, and the droplet formation process enters the detaching stage. A cylindrical neck forms between the floating droplet and the tip of the tube. The continuous growth of the droplet size results in a decrement in the curvature of the droplet, whereas the pressure variation inside and outside the droplet due to fluid flow is almost negligible. The Young–Laplace equation implies that the curvature of the neck parallel to the tip of the tube must be increased, leading to a decreasing radius of the neck. The pinching of the neck is attributed to the drastic increment of the droplet length as shown in Figure 5. Owning to the strict periodicity of the droplet formation process, highly monodispersed droplets can be produced through the dripping regime with a c.v. (coefficient of variation) in the radius lower than 0.1%.

**Figure 6:**
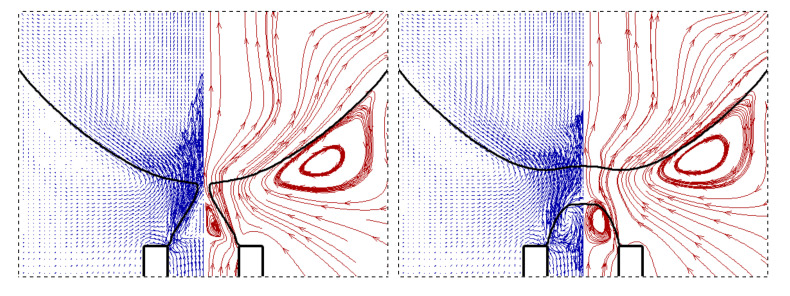
Velocity vectors (**left**) and streamlines (**right**) at the instant of droplet detaching (*q*_d_ = 90 mL/h, *We* = 0.027, *Bo* = 0.0038).

As presented in Figure 6, axisymmetric vortexes are observed inside the droplet due to the pinching motion of the neck. The streamlines are confined by the interface after the dispersed phase flows out of the tip, and thus the dispersed phase is forced to flow backward and the pressure at the upstream of the neck inside increases rapidly. The dispersed phase downstream of the neck is accelerated vertically towards the flow direction caused by the squeezing from the neck, which in turn helps the fast draining of the fluid inside the neck. Due to this local high velocity of the dispersed phase, the interface oscillates several times after detaching until the kinetic energy transforms to surface energy completely as plotted in the inset of Figure 5.

### 4.2. Jetting

**Figure 7:**
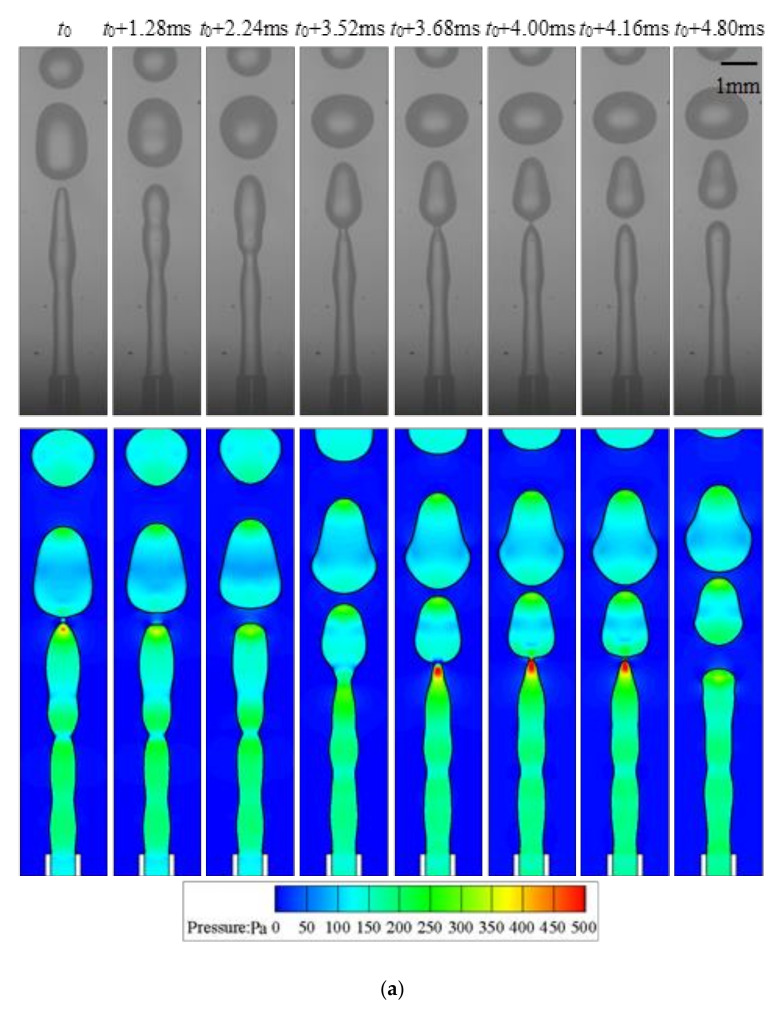

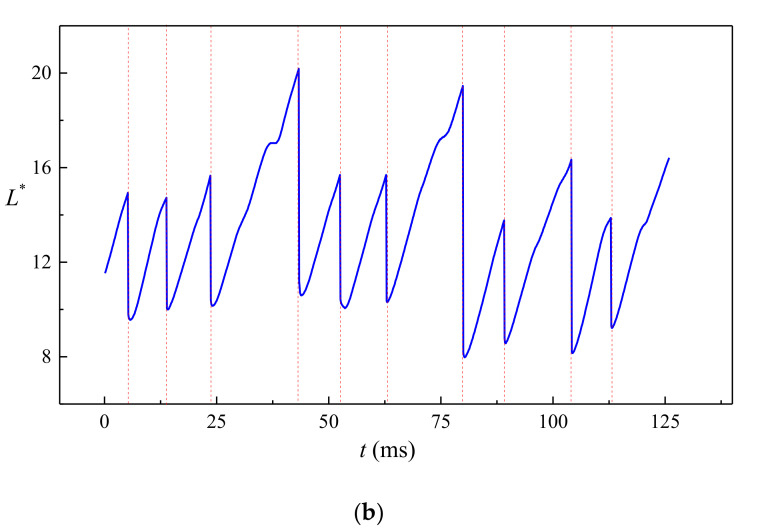
Typical jetting regime (*q*_d_ = 355 mL/h, *We* = 0.42, *Bo* = 0.0038): (**a**) experimental snapshots and numerical simulation; (**b**) variation of droplet length reconstructed from numerical simulation.

Jetting is another common regime distinct from dripping. In this case, the droplets are formed at the end of a jet, as illustrated in Figure 7. The jet does not retreat to the tube after the detachment of the droplets, and hence L* is always greater than 1. The behavior of the interface in the jetting regime is dominated by inertial force owing to the high velocity of the dispersed phase. The tube is nonwettable by the dispersed phase in all cases studies in this work, and the wetting radius of the jet is always *R*_i_. The variation of L* is almost linear without strict periodicity as summarized in Figure 7b. Fluctuations are observed on the interface due to the development of capillary wave, and the local pressure is affected by the unsmooth interface as shown in Figure 7. Like jetting regimes in other classes of microfluidic devices, the growing and detaching of the droplet are the result of the fastest-growing perturbation on the jet [46]. Compared to dripping, the volume of the detaching droplet is still growing when the neck is pinching. Though the pressure inside the interface increases upstream the pinching location, similar to what is observed in the dripping regime, no vortex motion is observed during detaching of the droplets. However, after the droplet is produced, the liquid thread must be smoothed, and hence transient vortex occurs at the front of the dispersed phase due to the strong retraction of the interface.

### 4.3. Dripping-to-Jetting Transition

**Figure 8:**
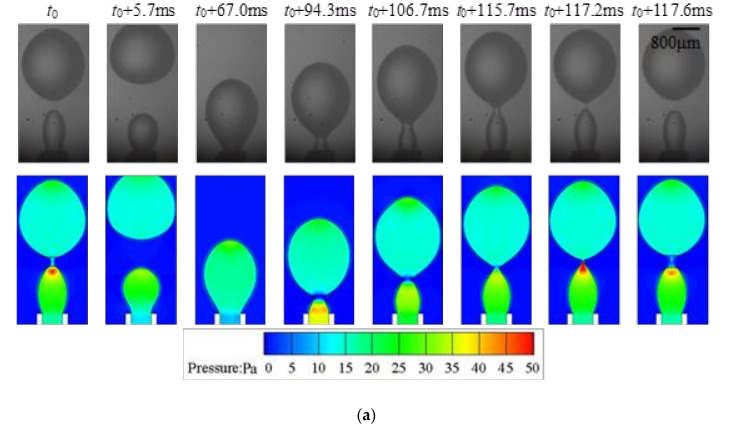

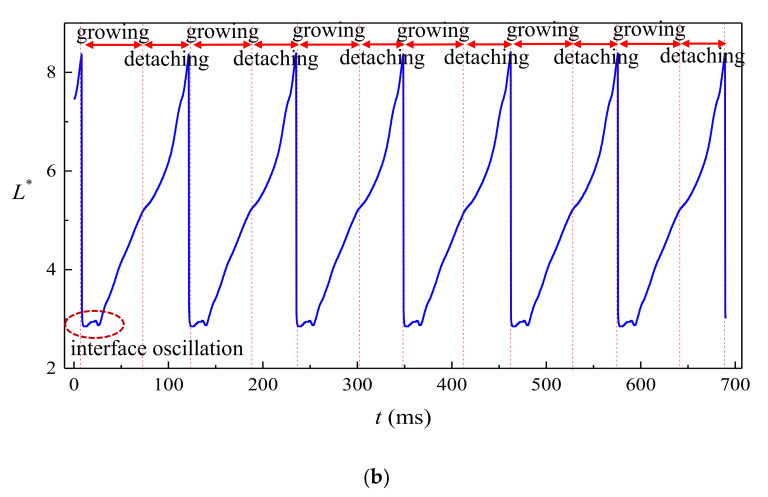
Typical transitional regime (*q*_d_ = 75 mL/h, *We* = 0.13, *Bo* = 0.027) with 0.5 wt% SDS in water: (**a**) experimental snapshots and numerical simulation; (**b**) variation of droplet length reconstructed from numerical simulation.

The transition from dripping to jetting can be triggered spontaneously by increasing the flow rate of the dispersed phase. However, the transition does not occur abruptly. Patterns, namely transitional regimes with characteristics of both the dripping and jetting regimes are observed (Figure 8). Like jetting, the dispersed phase does not retract to the tip entirely, and the remaining droplet at the tip is already deviated from spherical after the detachment of the previous droplet. The formation process of a droplet under the transitional regime is also divided into growing and detaching stages, similar to dripping regime, and the variation of L^*^ is accelerated in the detaching stage as well. However, the inertial force is much stronger compared to dripping, which results in a relatively shorter growing time (approximately 60% of the entire droplet formation time in Figure 8). A strong retraction of the front interface is also observed at the beginning of each droplet growing stage. However, the competition between interfacial tension and inertial force is intensified, and longer time (16% of the droplet formation time in the case shown in Figure 8) of oscillations is experienced until the kinetic energy can be transformed into surface energy.

**Figure 9:**
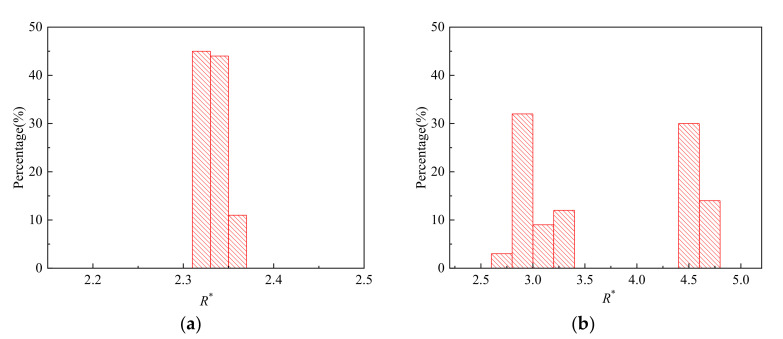
Size distribution of droplets: (**a**) *q*_d_ = 75 mL/h, *We* = 0.13, *Bo* = 0.027; (**b**) *q*_d_ = 255 mL/h, *We* = 0.22, *Bo* = 0.0038.

Two classes of transitional regimes are observed that produce monodispersed droplets (Figure 9a) and binary dispersed droplets (Figure 9b). Monodispersed droplets are produced when the formation processes of each droplets have strict periodicity similar to dripping and only one droplet is produced in each period. On the other hand, when the remaining dispersed phase on the tip after detaching varies between each droplet formation period, binary dispersed droplets are produced. The alternate detachment of bigger and smaller droplets is observed. For example, in the case shown in Figure 9b, a smaller droplet with R* ≈ 3.0 is produced, following which a bigger droplet with R* ≈ 4.6 is produced. Hence, 50% of the collected droplets are R* ≈ 3.0 while the other 50% are droplets with R* ≈ 4.6. It can be concluded from Figure 9b that the polydispersity of each type of droplet is still below 5%, which can potentially be useful in testing the performance of droplet screening devices.

### 4.4. Effect of Interfacial Tension Coefficient

Interfacial tension plays a critical role in the formation processes of droplets. To achieve adjustment in interfacial tension coefficient *σ*, SDS was dissolved in water and used as the continuous phase.

Under the condition of 0 SDS (*σ* = 49.6 mN/m) load the interfacial tension coefficient is relatively high and the dripping regime is observed in a wide range of the flow rate of the dispersed phase *q*_d_ (0–255 mL/h). Although slightly smaller droplets are produced when *q*_d_ increases, the variation of R^*^ is within 15% in the entire range of *q*_d_ covered by the dripping regime. The resulting statistics suggest that droplet size is insensitive to *q*_d_ under the dripping regime. Since higher droplet formation frequency is achieved under the condition of higher *q*_d_, increasing *q*_d_ while still maintaining the dripping regime is a reliable approach to increase the production rate in a buoyancy-assisted device. However, the range of *q*_d_ covered by dripping shrinks by 50% when *σ* is reduced to 20.5 mN/m with 0.1 wt% of SDS added in the continuous phase. Eventually, dripping is observed only in the range of *q*_d_ ≈ 0–60 mL/h when the concentration of SDS is increased to 0.25 wt% and *σ* is reduced to 7.4 mN/m. Obviously, the interfacial tension is reduced greatly by SDS and less inertial force is required to cause the transition of dominate force.

**Figure 10:**
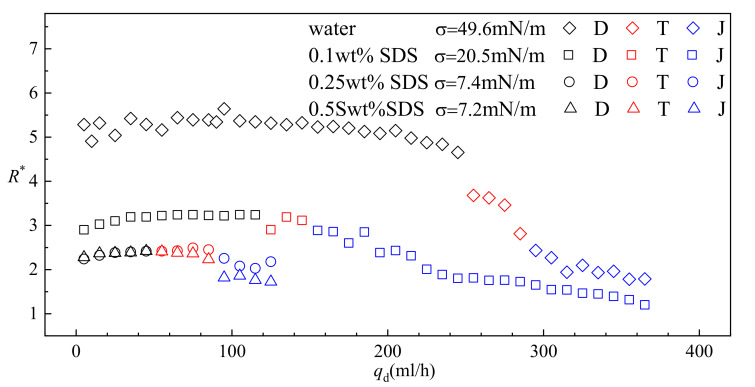
Effect of the interfacial tension coefficient on droplet size and formation regimes (D denotes dripping regime, T denotes transitional regime and J denotes jetting regime).

As *q*_d_ is increased further, stronger inertial force is induced. Under the condition of 0 SDS load, a drastic decrement in R^*^ is observed in a relatively narrow range of *q*_d_ (225–295 mL/h) during which the transitional regime occurs. Usually, this noncontinuous variation in R* during the increment of *q*_d_ is identified as the result of inertial force dominance over interfacial tension, and the flow patterns in this range of *q*_d_ are classified as jetting [36]. However, after examining the dynamics of the fluids combining both experiment and numerical results, we identified this as a transitional regime since the interfacial tension is not dominated by the inertial force completely. Moreover, some behaviors of the fluids, such as the vortexes upstream the draining neck during detaching, imply a strong effect of interfacial tension. Surprisingly, the variation of R^*^ is depressed in systems with lower *σ,* as shown in Figure 10. Especially of note, droplets produced under dripping and transitional regime are almost the same size when *σ* = 7.4 mN/m. This can be attributed to a reduced net force caused by the reduction in interfacial tension. R^*^ continues decreasing when stable jetting is developed after inertial force dominates over interfacial tension.

It is worth noting that the absorption of SDS onto the interface influences the interfacial tension not only statically but also dynamically [47,48,49,50,51]. Molecules of SDS are absorbed onto the interface as the interface area increases during droplet formation [49]. In Figure 10, 0.5 wt% SDS is over the CMC (critical micelle concentration) of SDS in DI water. A difference in droplet size produced in the jetting regime is observed between the results from systems using 0.25 wt% SDS and 0.5 wt% SDS. Since the evolution of the interface is much slower in dripping regime than that in jetting regime, the absorption of SDS is fast enough to form an interface with homogenous interfacial tension. Hence, no difference in droplet size produced in dripping regime is observed between systems using 0.25 wt% SDS and 0.5 wt% SDS. As for the transitional regime, when the flow rate is low, the influence of varying dynamic interfacial tension is similar to that observed during dripping. With increasing flow rate, smaller droplets are produced in the system using 0.5 wt% SDS, similar to jetting.

In addition, the droplet size is expected to vary when using different types of surfactant under the same flow rate. Smaller droplets will be produced in a system with lower static interfacial tension. The variation of droplet size and formation regime with *q*_d_ is expected to follow a tendency similar to that of a system using SDS. For systems that use surfactant with large molecules and slow absorption rate, the influence of dynamic interfacial tension will be stronger than that observed in the cases studied in this work.

### 4.5. Regime Diagram

**Figure 11:**
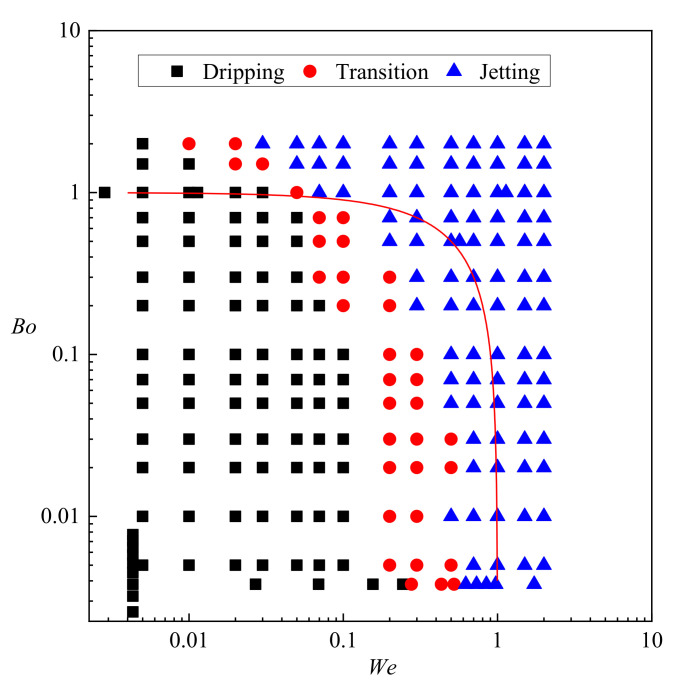
Regime diagram in the coordinates of *We* and *Bo.*

To provide a systematic view of the regimes in the buoyancy-assisted microfluidic device, a regime diagram is summarized from the results of both experimental observation and numerical simulation in Figure 11. As defined in Equations (13) and (14), *We* and *Bo* describe the relationship between inertial force, buoyancy and interfacial tension, and the solid red line in Figure 11 is *We* + *Bo* = 1. Obviously, most dripping cases are limited to the conditions when *We* + *Bo* is less than 1, where the net force (sum of the inertial and buoyancy force) driving the droplet away from the tip is smaller than the interfacial tension. Surprisingly, the droplets are still formed under dripping regime even when *Bo >* 1 as long as *We* is kept small enough (*We* ≤ 0.01 in Figure 11). In these cases, even though *We* + *Bo* is larger than 1, the jetting regime or even the transitional regime cannot be triggered. On the other hand, most jetting regimes are limited to the conditions of *We* + *Bo >* 1. The regime diagram suggests that regardless of *Bo*, the transitional regime is developed provided *We* > 0.2, and the jetting regime is developed provided *We* > 0.5. It can be concluded that sufficient inertial force is an indispensable condition to trigger the transition from dripping to jetting. Besides, unlike other microfluidic devices where widening jetting and thinning jetting are observed [52], only one class of jetting exists in the buoyancy-assisted microfluidic device, and the droplet radius is always larger than jet radius. Since the radius of the jet is approximately the inner radius of the tube, producing droplets with radius smaller than the tube cannot be achieved in a buoyancy-assisted device. Note that the droplet formation is influenced by the surfactant type, especially by absorption rate of the surfactant. In systems that use different types of surfactant with different absorption rates, the range covered by dripping and transitional regime in Figure 11 is expected to be different.

## 5. Conclusions

A comprehensive study focusing on the droplet formation process in a buoyancy-assisted microfluidic device was conducted, combining the effort of visualization experiment and numerical simulation. Typical regimes, including dripping and jetting were studied to elucidate their characteristics. Of special interest, the transition from dripping regime to jetting regime was examined to provide an in-depth understanding of the transitional behaviors. The effect of interfacial tension coefficient on the transition behavior from dripping to jetting was discussed. Finally, a regime diagram was summarized to help design buoyancy-assisted devices and corresponding control strategies.

Major conclusions of this work are as follows:

(1) Strong periodicity is observed in the dripping regime where highly monodispersed droplets can be produced with c.v. in radius below 0.1%. The growing stage occupies over 90% of the time in one droplet formation period. During the detaching stage, axisymmetric vortexes are observed inside the droplet caused by reversed flow of the dispersed phase near the axis. The dispersed phase downstream of the neck is squeezed and accelerated towards the flow direction, resulting in fast draining of the dispersed phase inside the neck.
(2) The droplet length L^*^ varies almost linearly with time in each droplet formation period in the jetting regime. Fluctuations on the interface are observed due to the development of the capillary wave. Droplets produced in the jetting regimes in buoyancy-assisted devices are always larger than the inner radius of the injection tube.
(3) Transition regimes with the characteristics of both dripping and jetting are observed when Weber number *We* is increased. The duration of the growing state in a transitional regime is shortened compared to dripping.
(4) The sizes of the droplets are insensitive to the flow rate *q*_d_ in dripping regimes. Reducing interfacial tension coefficient results in narrower range of *q*_d_ that is covered by dripping. Moreover, the transition from dripping to jetting can be triggered at lower *q*_d_.
(5) The regime diagram suggests that most dripping cases occur when *We* + *Bo* < 1 while most jetting cases occur when *We* + *Bo* > 1. However, only sufficient inertial force from the dispersed phase is the indispensable condition of triggering the transition behavior from dripping to jetting. Even when buoyancy dominates over interfacial tension (*Bo* > 1), the droplets are still formed under dripping regime as long as *We* < 0.01. On the other hand, the formation regime is always jetting when *We* > 0.5 regardless of *Bo*.

## Figures and Tables

**Table 1:** The physical properties of all the fluids.

Material	Density *ρ* (kg/m^3^)	Interfacial Tension Coefficient *σ* (mN/m)
n-octane	702	
DI water	998.2	49.6
0.1 wt% SDS in DI water	998.2	20.5
0.25 wt% SDS in DI water	998.2	7.4
0.5 wt% SDS in DI water	998.2	7.2
